# No indication of histological changes in embryonic somatosensory cortex development upon maternal aspartame consumption in mice

**DOI:** 10.1038/s41538-025-00679-2

**Published:** 2026-01-08

**Authors:** Liliia Andriichuk, Takashi Namba

**Affiliations:** 1 Neuroscience Center, HiLIFE – Helsinki Institute of Life Science, University of Helsinki, Helsinki, Finland; 2 Department of Developmental Biology, Fujita Health University School of Medicine, Toyoake, Japan; 3 Division of Developmental Neurobiology, International Center for Brain Science (ICBS), Fujita Health University, Toyoake, Japan

**Keywords:** Developmental biology, Neuroscience

## Abstract

Non-nutritive sweeteners are widely used in multiple diets and are considered a healthy alternative for pregnant women reducing sugar consumption, thereby preventing maternal obesity and gestational diabetes. While some controversies have been raised regarding their safety for human consumption, particularly aspartame, the effects of aspartame on prenatal brain development have rarely been studied. In this study, we investigated whether maternal aspartame consumption within a physiologically relevant range for normal daily consumption by humans affects neocortical development in mice. Here we show that daily maternal aspartame consumption at 18% of the European Food Safety Authority-approved daily dosage does not significantly alter the structure of the somatosensory cortex, nor the numbers of excitatory neurons, inhibitory neurons, astrocytes, or oligodendrocytes in mouse pups less than 48 hours old. These results suggest that aspartame has no detectable impact on somatosensory cortex development in mice. This study provides additional information that can be utilized by expectant mothers making choices about their diet.

## Introduction

Pregnancy causes multiple changes in the maternal body. One well-known change is the establishment of new eating behaviors, which could lead to conditions such as maternal obesity and gestational diabetes^1^. Maternal food intake impacts the embryo/fetus directly and can affect postnatal development leading to the emergence of metabolic disorders in adulthood^2,3^. To avoid such adverse effects, a significant proportion of expectant mothers try to limit daily consumption of sugar, and in exchange, consume more non-nutritive sugar substitutes, that is, sweeteners.

Over the last few decades, the number of mass-produced food products containing non-nutritive sweeteners, such as sugar-free chewing gum, diet beverages, sweets, dairy products, and sports nutrition has been increased. Although all sweeteners on the market have been approved by the authorities, for example, the Food and Drug Administration (FDA) in the United States and European Food Safety Authority (EFSA), some controversies have been raised regarding their safety for human consumption^4,5^. One of the most controversial sweeteners is aspartame, which is 180-200 times sweeter than sucrose. Aspartame is metabolized and converted into aspartic acid (40%), phenylalanine (50%), and methanol (10%), which is further broken down into formaldehyde and formic acid^6,7^. There are a limited number of studies investigating the impact of aspartame consumption on human maternal health, pregnancy, and embryo/fetus development. The latest systemic review with a meta-analysis of 19 studies on the impact of aspartame during pregnancy concluded that sweeteners, mainly aspartame, are associated with an 11% increased risk of early preterm delivery in expectant mothers^8^, suggesting that maternal aspartame consumption possibly affects the development of the fetus. While previous studies indicate that aspartame has a negative effect on the placenta in mice^9^, the effects of aspartame on fetal brain development have rarely been studied.

The neocortex, a brain region responsible for higher cognitive functions, is developed in a tightly regulated manner^10–12^. The neural stem/progenitor cells (NPCs) residing in the ventricular zone (VZ) and the subventricular zone (SVZ) first generate neurons which migrate into the cortical plate (CP) to eventually form the six-layered neocortex. Neurogenesis, the production of neurons, is followed by macroglial cell production, also known as gliogenesis. This developmental process is known to be sensitive to external disruptors such as ethanol^13–15^. Maternal intake of such disruptors results in changes in neurogenesis and gliogenesis, leading to impaired neocortex development, is the basis of abnormal brain function observed later in life^16,17^.

In this study, we investigated whether maternal aspartame consumption affects neocortical development in mice, with a particular focus on neurogenesis, gliogenesis and neuronal layer formation, which are known to be largely completed during the embryonic and perinatal periods. To this end, we focused on the somatosensory cortex as a representation of mouse neocortex. We showed that daily maternal aspartame consumption at 18% of the EFSA-approved daily dosage, a value within range of actual average daily consumption^18^, did not significantly alter the structure of the somatosensory cortex in pups after delivery. Our data suggest that maternal aspartame consumption, at least at the dose used in the present study, does not significantly disrupt the proliferation and differentiation of progenitor cells, and the layer formation of neurons within the somatosensory cortex of newborn mice. This study provides additional information that can be utilized by expectant mothers making choices about their diet.

## Results

### Undetectable changes in the weight of pups after maternal aspartame consumption

**Fig. 1:**
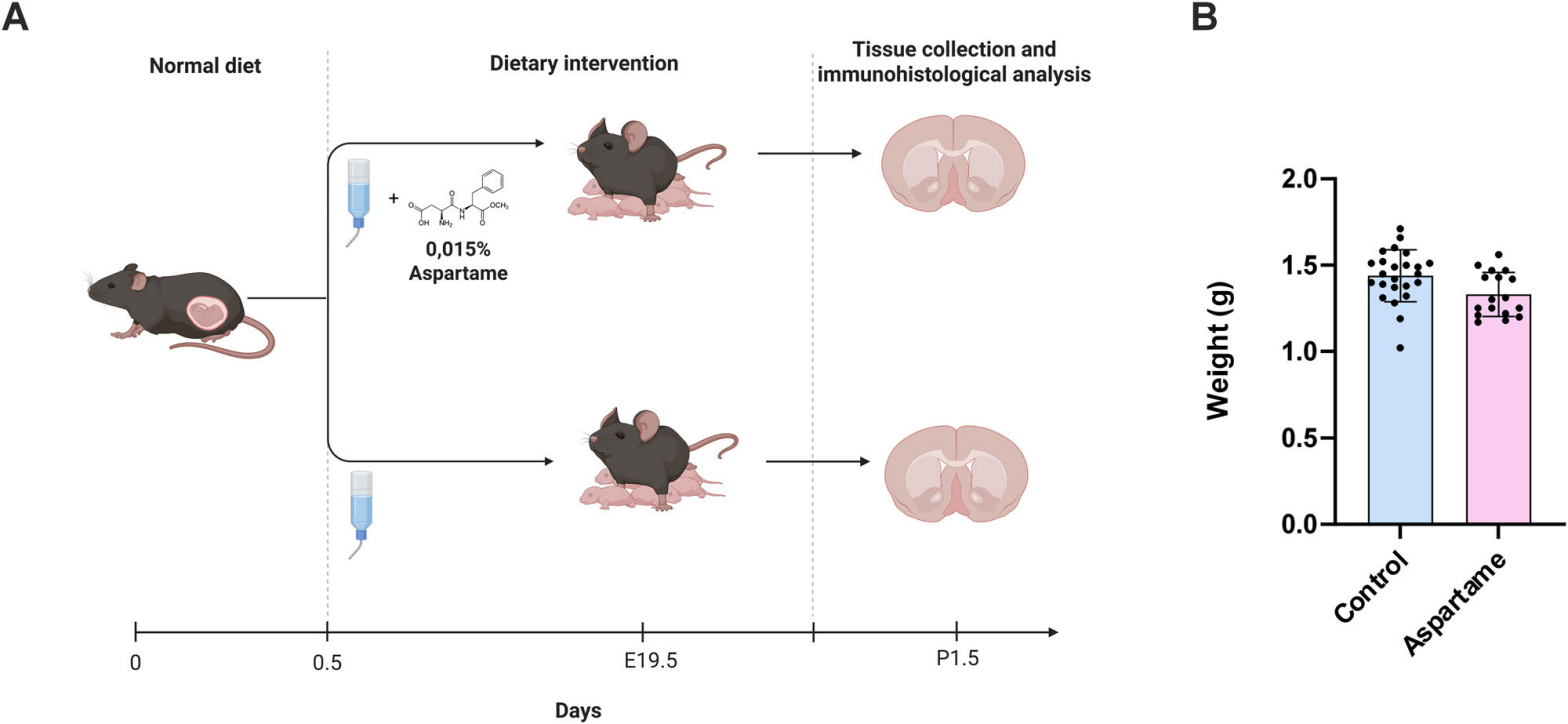
Experimental design and undetectable changes in weights of aspartame pups. **A** Schematic illustration of the timeline of the experiment. Pregnant dams were given water or water with 0.015% aspartame from E0.5 to the pups’ birth and tissue was collected for immunohistological analysis at P1.5. Created in BioRender. Andriichuk, L. (2025) https://BioRender.com/fypir8k. **B** Weight of control (n = 24, 3 dams) and aspartame (n = 17, 2 dams) pups at P1.5. Mean ± SD. Unpaired t-test.

To study the impact of aspartame exposure on embryonic development, we introduced drinking water containing 0.015% aspartame to adult female mice starting on the day of gestation (referred to as embryonic day 0.5 (E0.5)) (Fig. 1A). The concentration of aspartame in drinking water was based on previous reports^19^. The dams received either drinking water without aspartame (control) or drinking water with aspartame during the whole gestational period. All dams delivered at E19.5 and pups from control mothers (hereafter referred to as control pups) and the pups from the aspartame-consuming mothers (hereafter referred to as aspartame pups) were weighed at postnatal day 1.5 (P1.5). We did not observe any significant changes in weight between control and aspartame pups (Fig.1B), suggesting that maternal aspartame consumption does not influence the weight of pups at early postnatal stages. We did not observe any significant difference in the daily maternal consumption of water between groups (Supplementary Fig. 1). The amount of daily aspartame consumption per body weight of the mice was within range of estimated daily consumption per body weight in adult humans^20,21^.

### Influences of maternal aspartame consumption on embryonic somatosensory cortex development

To examine the effect of maternal aspartame consumption on somatosensory cortex development in embryos, we analyzed the main characteristics of somatosensory cortex development in mouse pups at P1.5. This time point represents the completion of the major prenatal events, including neurogenesis and neuronal layer formation, while preceding the peak period of complex postnatal events, such as dendritic arborization and activity-dependent synaptic remodeling. In this study, we analyzed the following five aspects: i) layer formation, ii) excitatory neurons, iii) interneurons, iv) macroglia and their progenitors and v) apoptosis.

### Somatosensory cortex layer formation

**Fig. 2:**
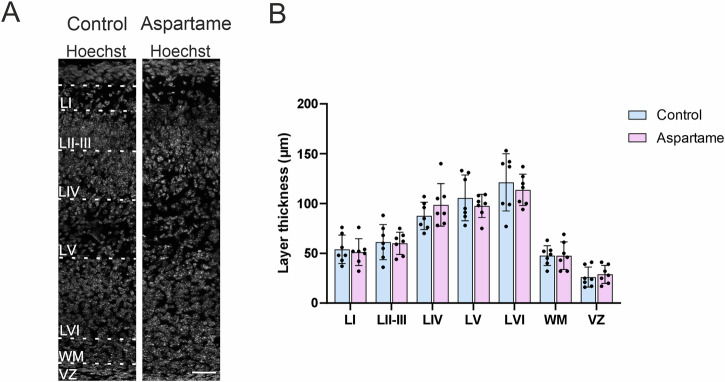
No significant changes in the cortex thickness between control and aspartame pups. **A** Representative images of neocortices at P1.5. The layers were distinguished by Hoechst staining. LI: layer 1, LII/III : layers 2 and 3, LIV: layer 4, LV: layer 5, LVI: layer 6, WM: white matter and VZ: ventricular zone. **B** The thickness of each layer (based on Hoechst staining) in the neocortex of control (n = 7 brains, 3 dams) and aspartame (n = 7 brains, 2 dams) pups. Mean ± SD. Mann-Whitney U test. Scale bar: 50 µm in (**A**).

Histological changes in somatosensory cortex development upon maternal aspartame consumption, if any, could be reflected as alterations in the six-layer structure. In light of this notion, we first examined somatosensory cortex layer formation at P1.5. To this end, the thickness of each layer was measured in the somatosensory cortex. The layer structure was visualized by nuclear staining with Hoechst. There were no significant differences in the thickness of each neocortical layer between control pups and the aspartame pups (Fig. 2A, B), suggesting that the overall structure of the somatosensory cortex was not affected by maternal aspartame consumption at the present dose.

### Excitatory neurons

**Fig. 3:**
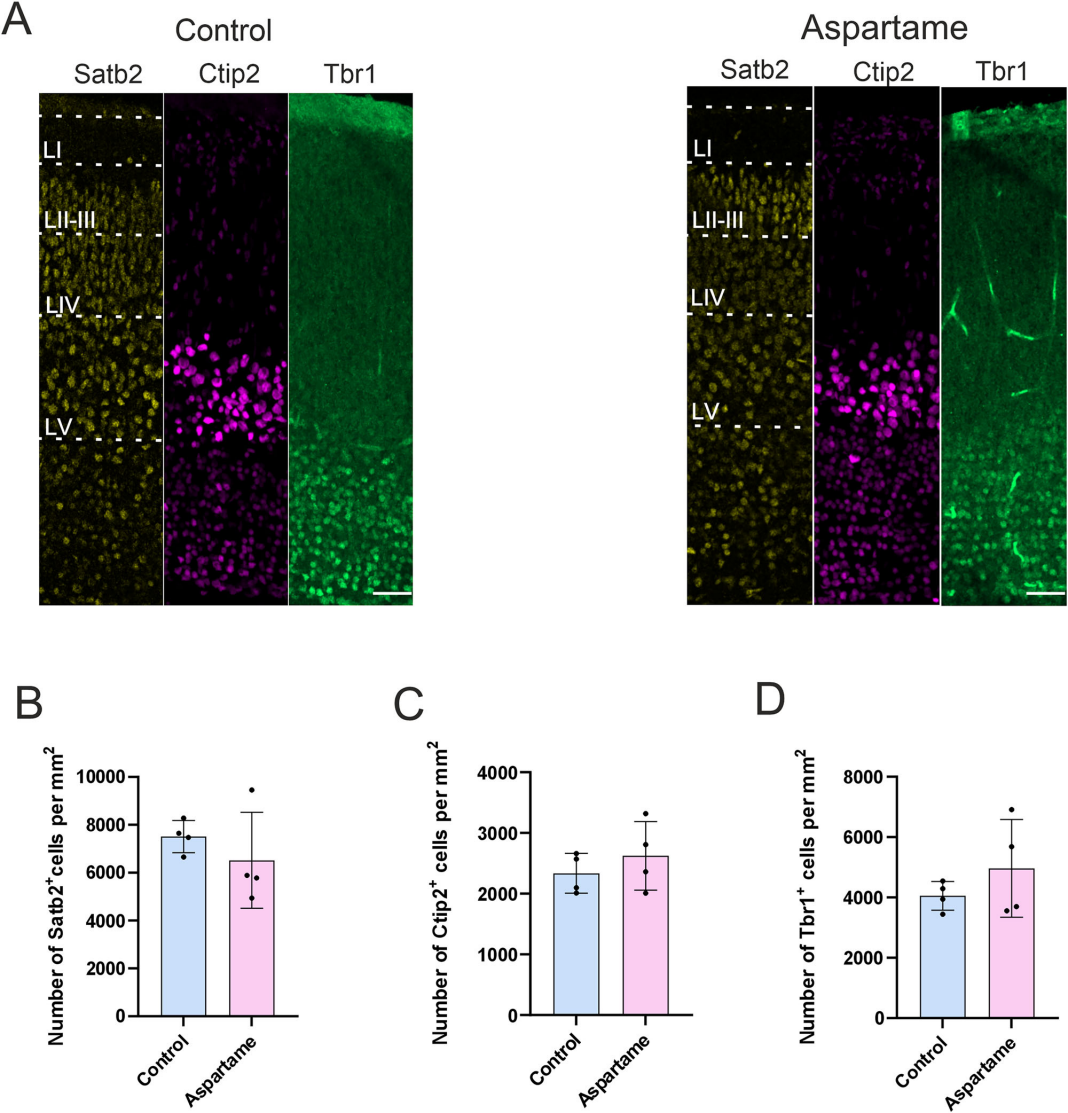
No changes in neurons expressing neocortical layer-markers in control and aspartame pups. **A** Representative images of Satb2 (yellow)-, Ctip2 (magenta)- and Tbr1 (green)-positive cells. **B** Quantification of Satb2-positive (Satb2^+^) cells from control (n = 4 brains, 3 dams) and aspartame (n = 4 brains, 2 dams) pups. **C** Quantification of Ctip2-positive (Ctip2^+^) cells from control (n = 4 brains, 3 dams) and aspartame (n = 4 brains, 2 dams) pups. **D** Quantification of Tbr1-positive (Tbr1^+^) cells from control (n = 4 brains, 3 dams) and aspartame (n = 4 brains, 2 dams) pups. LI: layer 1, LII/III: layers 2 and 3, LIV: layer 4, LV: layer 5, LVI: layer 6. Mean ± SD. Mann-Whitney U test. Scale bars: 50 µm in (**A**).

The somatosensory cortical layer structure is mainly determined by the number and localization of distinct subtypes of excitatory neurons. Therefore, to corroborate the layer structure analysis described above, we evaluated the abundance of excitatory neurons. We quantified the number of excitatory neurons expressing the following layer-specific marker proteins: Satb2 for the layer II-IV, Ctip2 for the layer V and Tbr1 for the layer VI^22–25^. The number of Satb2-positive neurons was not significantly different, albeit there is a trend in reduction, between control pups and aspartame pups (Fig. 3A, B). Similarly, no noticeable changes were observed in neither the number of Ctip2-positive (Fig. 3A, C) and Tbr1-positive (Fig. 3A, D) neurons, nor the localization of neurons expressing each marker protein between the two groups. These results indicate that maternal aspartame consumption at the present dose does not affect the neuronal layer formation of the embryonic neocortex in general.

### Interneurons

**Fig. 4:**
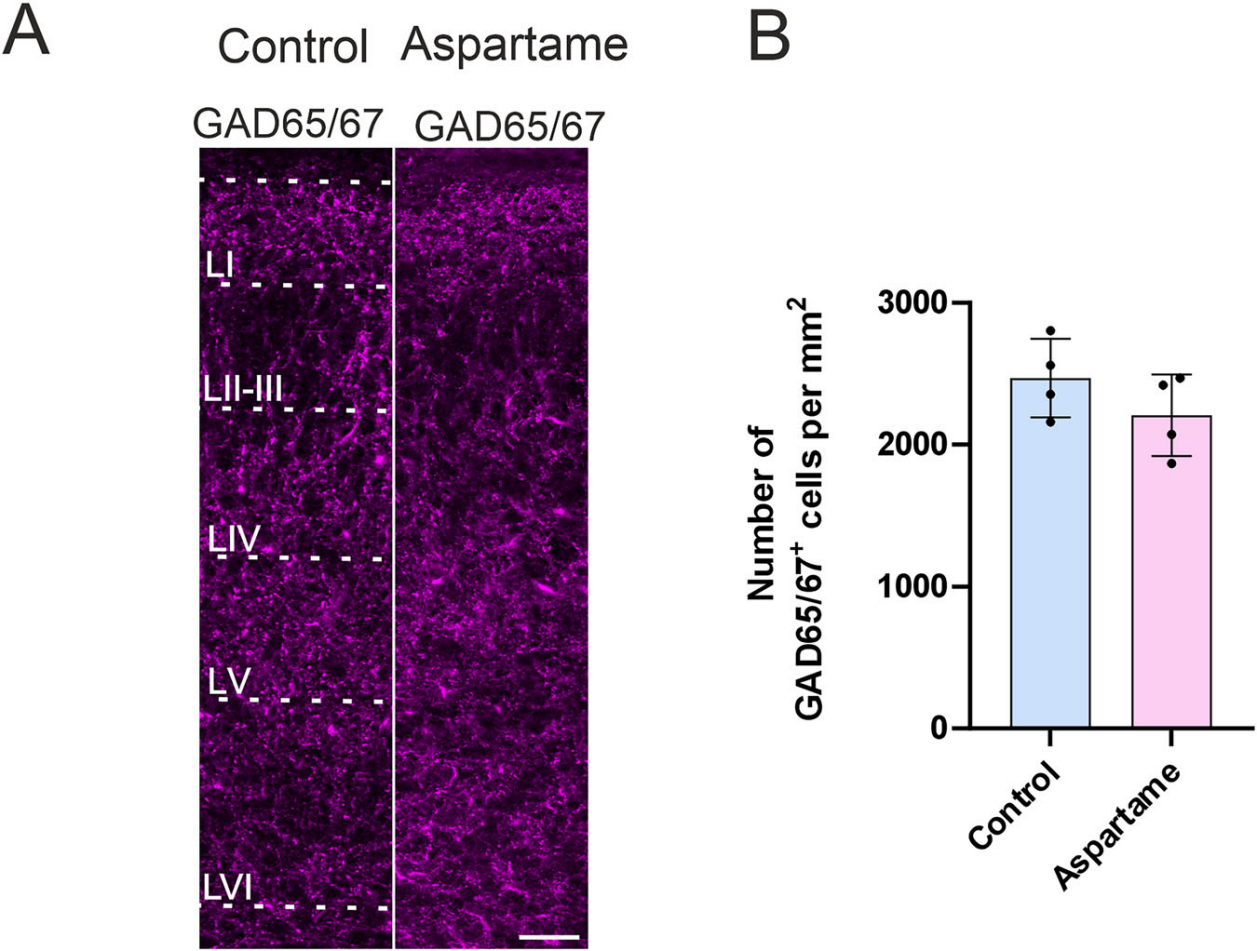
No changes in neurons expressing GAD65/67 markers in control and aspartame pups. **A** Representative images of GAD65/67 (magenta)-positive cells. **B** Quantification of GAD65/67-positive (GAD65/67^+^) cells from control (n = 4 brains, 3 dams) and aspartame (n = 4 brains, 2 dams) pups. LI: layer 1, LII/III: layers 2 and 3, LIV: layer 4, LV: layer 5, LVI: layer 6. Mean ± SD. Mann-Whitney U test. Scale bar: 50 µm in (**A**).

Interneurons, mainly GABAergic neurons, are an essential component of the somatosensory cortex. The number of interneurons needs to be tightly regulated, otherwise the neuronal network in the neocortex will exhibit abnormal firing patterns, which for example, leads to epileptic neuronal activity^26,27^. To examine the abundance of interneurons in the somatosensory cortex, we quantified the number of glutamic acid decarboxylase (GAD65/67)-expressing cells and found that the number of the GAD65/67-positive cells was not changed between control pups and aspartame pups (Fig. 4A, B).

### Macroglia and their progenitors

Macroglia, astrocytes, and oligodendrocytes are known to be generated from gliogenic progenitor cells in the late embryonic to neonatal periods. The gliogenic progenitor cells are known to express the marker Olig2^28,29^ and the proliferating cell marker Ki67^30,31^. The Olig2-positive gliogenic progenitor cells give rise to astrocytes that express S100b. The immature astrocytes express both Olig2 and S100b, but mature ones only express S100b. A subset of Olig2-positive gliogenic progenitor cells, which are committed to producing oligodendrocytes, express Sox10^31^. Since expression of Olig2 and Sox10 persists during their maturation, Olig2- and Sox10-double positive cells are considered oligodendrocyte progenitors and postmitotic oligodendrocytes.

**Fig. 5:**
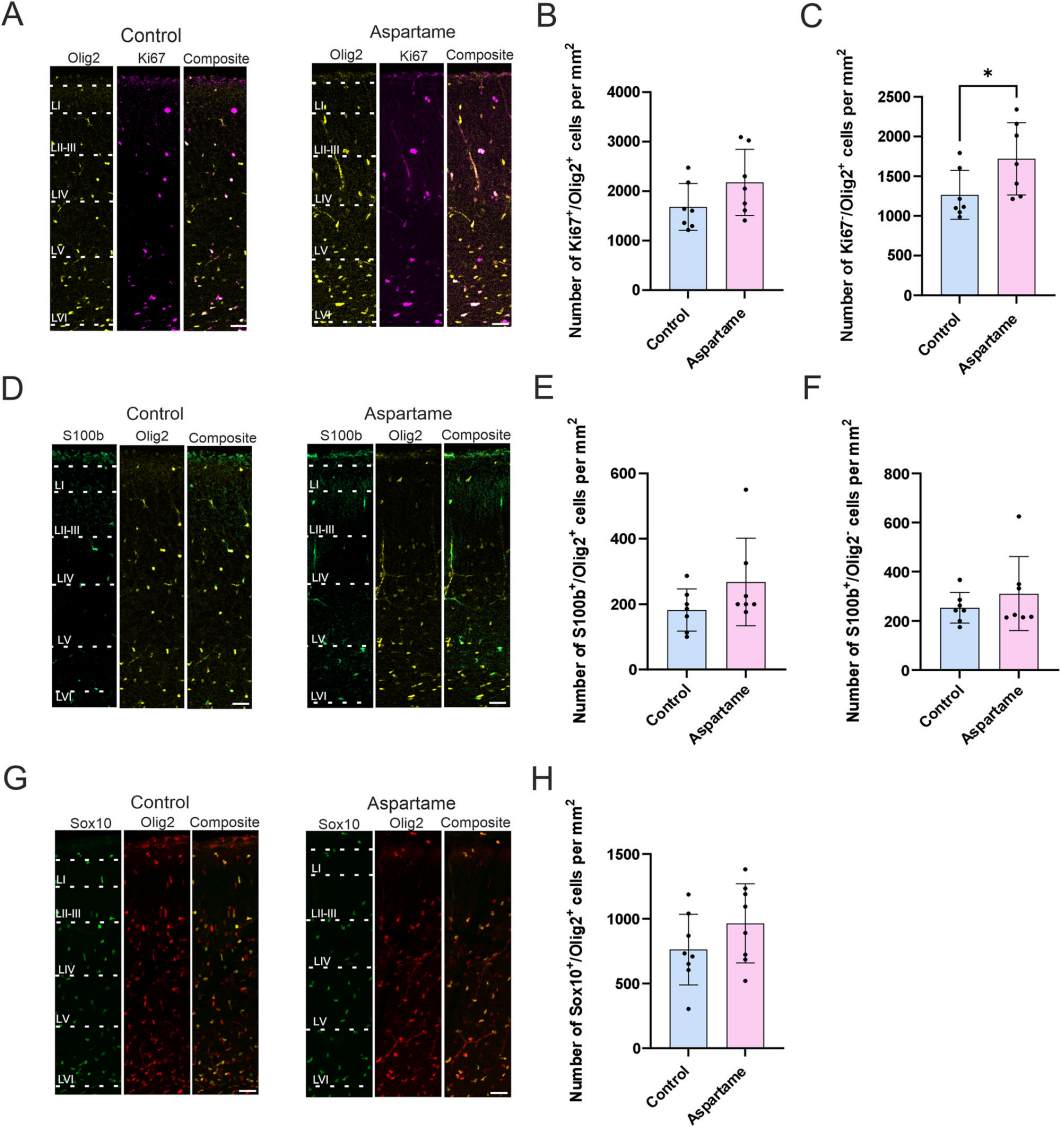
No differences in gliogenic progenitors, astrocytes, or oligodendrocytes in control and aspartame pups. **A** Representative images of Olig2 (yellow)- and Ki67 (magenta)-positive (Ki67^+^/Olig2^+^) cells. **B** Quantification of Ki67^+^/Olig2^+^cells from control (n = 7 brains, 3 dams) and aspartame (n = 7 brains, 2 dams) pups. **C** Quantification of Ki67-negative and Olig2-positive (Ki67^-^/Olig2^+^) cells from control (n = 7 brains, 3 dams) and aspartame (n = 7 brains, 2 dams) pups. **D** Representative images of S100b (green) and Olig2 (yellow)-positive (S100b^+^/Olig2^+^) cells. **E** Quantification of S100b^+^/Olig2^+^ cells from control (n = 7 brains, 3 dams) and aspartame (n = 7 brains, 2 dams) pups. **F** Quantification of S100b-positive and Olig2-negative (S100b^+^/Olig2^-^) cells from control (n = 7 brains, 3 dams) and aspartame (n = 7 brains, 2 dams) pups. **G** Representative images of Sox10 (green)- and Olig2 (red)-positive (Sox10^+^/Olig2^+^) cells. **H** Quantification of Sox10^+^/Olig2^+^ cells from control (n = 7 brains, 3 dams) and aspartame (n = 8 brains, 2 dams) pups. LI: layer 1, LII/III: layers 2 and 3, LIV: layer 4, LV: layer 5, LVI: layer 6. Mean ± SD. Mann-Whitney U test (B, C, E, F; * *p* < 0.05) and unpaired Student’s t-test (**H**). Scale bars: 50 µm in (**A**), (**D**), and (**G**).

First, we examined the abundance of gliogenic progenitor cells. While the number of Olig2- and Ki67-double positive gliogenic progenitor cells was not significantly changed between control pups and aspartame pups, albeit there is a trend of an increase (Fig. 5A, B), the number of postmitotic glial cells expressing Olig2 but not Ki67 was slightly increased upon maternal aspartame consumption (Fig. 5A, C). However, there were no changes in the number of Olig2 and S100b-double positive cells (Fig. 5D, E) nor Olig2-negative and S100b-positive cells (Fig. 5D, F). The number of Olig2 and Sox10-double positive cells was not changed between the two groups (Fig. 5G, H). Taken together, these data suggest that the abundance of astrocyte-committed cells and oligodendrocyte-committed cells was not affected by maternal aspartame consumption at the present dose.

### Apoptosis

**Fig. 6:**
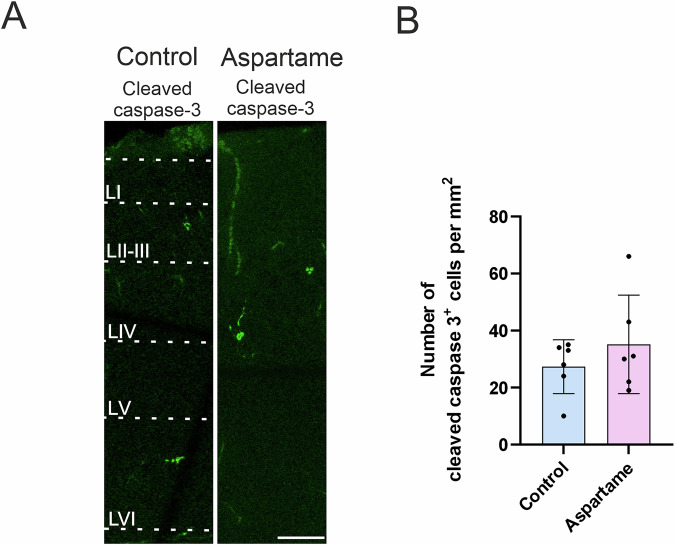
No changes in apoptosis in control and aspartame pups. **A** Representative images of cleaved caspase-3 (green) -positive cells. **B** Quantification of cleaved caspase-3^+^cells from control (n = 6 brains, 3 dams) and aspartame (n = 6 brains, 2 dams) pups. LI: layer 1, LII/III: layers 2 and 3, LIV: layer 4, LV: layer 5, LVI: layer 6. Mean ± SD. Mann-Whitney U test. Scale bar: 50 µm in (**A**).

Lastly, we examined whether maternal aspartame consumption influences cell viability in the somatosensory cortex. To this end, we immunohistochemically analyzed the activation of caspase 3, an apoptosis inducer^32^. The number of cells expressing the activated form of caspase 3, that is, cleaved caspase 3, was not significantly altered upon maternal aspartame consumption (Fig.6). This result is consistent with the above-mentioned results which showed no reduction in the number of neurons or macroglia. Therefore, these results suggest that the level of apoptosis, and thus cell viability, was not affected by maternal aspartame consumption at the present dose.

## Discussion

Maternal metabolic changes often manifest as new dietary patterns during pregnancy^33^. As an alternative to sugar, consumption of non-nutritive sugar substitutes has been increasing in recent years^34^. However, little is known about its effect, if any, on prenatal brain development. Therefore, we exposed pregnant mice to doses of aspartame equivalent to 18% of the EFSA-approved human maximum daily consumption, which is equal to drinking 3 cans of 0.33 l diet soda for an adult human. In preclinical rodent models, aspartame and its catabolized metabolites have been linked to various neurochemical effects in the central nervous system^35–37^. Furthermore, aspartame exposure at 8-15% of FDA-approved maximum daily dosages (50 mg/kg body weight/day) led to increased anxiety and deficits in learning and memory in both female and male mice^21^. Maternal aspartame consumption within the approved EFSA-approved maximum amount is known to, but does not always, reduce growth rate of embryos^9^. Despite these adverse effects, the potential risk of maternal aspartame consumption, especially at the level of normal daily consumption in the real world, on prenatal brain development has not been well elucidated.

Even though maternal aspartame does not reach embryos^38^, its derivatives aspartate, phenylalanine, and methanol could cross the placental barrier. Aspartate is an excitatory neurotransmitter as well as a key metabolite for neural progenitor cells^39^. Phenylalanine is a causal metabolite of phenylketonuria due to a lack of phenylalanine catabolism. Patients with phenylketonuria may have intellectual disability, which is largely avoidable through a diet with lower phenylalanine. Methanol has well-known neurotoxicity^40^. However, at the level of the normal daily aspartame consumption in the real world, and even at the maximum level of the EFSA-approved consumption, the amount of these metabolites in the blood is known to be considerably lower than the amount inducing some toxicity^18^.

In the present study, we focused on the somatosensory cortex and examined the effect of maternal aspartame consumption on embryonic somatosensory cortex development of mice. Neocortical development consists of two distinct events: neurogenesis and gliogenesis. The outcome of these events is reflected in the number of neurons and macroglial cells (astrocytes and oligodendrocytes) in the neonatal period. We observed no overall changes at P1.5 in the thickness of somatosensory cortex, nor in the number of neurons and macroglia, upon maternal aspartame consumption, suggesting that the concentration of aspartame in our study has very little influence on somatosensory cortex development at this time point. Interestingly, the only change we did observe was a slight increase in the abundance of postmitotic Olig2-positive cells. Olig2 is a driver of astrocyte and oligodendrocyte development in the neocortex^41^, and ectopic upregulation of Olig2 expression leads to suppression of neurogenesis^42^. However, there were no significant changes in the abundance of Sox10-positive oligodendrocyte-lineage committed cells, S100b-positive astrocytes, or neurons. Although misexpression of Olig2 is reported to induce apoptosis in the mouse embryonic neocortex^42^, we did not observe a significant increase in the number of apoptotic cells. These results suggest that the slight changes in postmitotic Olig2-positive cells could be negligible in terms of neurogenesis and gliogenesis.

No detectable changes in the developmental events leading to large structural anomalies of brain upon maternal aspartame consumption suggest that its effect on brain development, if any, might be detectable at a finer level. For example, the following aspects need to be addressed in the future studies: i) Neuronal morphology, including synaptic formation, ii) balance of excitatory vs. inhibitory synaptic inputs, iii) gene and protein expressions, especially receptors and transporters, iv) responses of cells to external stimuli such as infection and v) neuromodulatory systems.

It is important to note that the observations in this work are most likely independent of the sweet receptor, because aspartame does not taste as sweet for most rodent models as it does for humans^43,44^. This lack of sweet taste is reflected in the nonsignificant difference in the water consumption by mice between the aspartame group and the control group, which is consistent with previous observations^19,45^. Our study focused on the somatosensory cortex since this neocortical area has been studied well in terms of its development^46–48^. Although the present results indicate that there are no prominent changes in neurogenesis, gliogenesis, neuronal layer formation, and apoptosis in the somatosensory cortex upon maternal aspartame consumption, we can not rule out the following possibilities: i) There might be subtle changes that have not been detected in the present study due to the limited sample size, and ii) there might be a possibility to have changes in such developmental events in other brain areas, such as the entorhinal cortex, the parietal cortex, and the frontal cortex^21^. Direct extrapolation to human subjects would require more analysis on metabolic equivalence between mice and humans. The present study has not addressed sex-specific changes in the brain development examined at P1.5, since we have randomly selected pups for analysis to have an assumption, in which the sex of the analyzed pups was not biased to one sex. In addition, long-term neurodevelopmental effects in mice need to be addressed to translate the results to human subjects.

In conclusion, maternal aspartame consumption up to 37 mg/kg/day in a mouse model, shows no indication of abnormal neurogenesis, gliogenesis in the somatosensory cortex.

## Methods

### Mice

All experiments were performed in accordance with the Directive 2010/63/EU and approved by the National Animal Experiment Board, Finland (license number ESAVI/14992/2024). Animals were housed in enriched IVC cages with a 12-hour light/12 hour dark cycle and given access to water and food ad libitum. 6 weeks old C57BL/6JRj (Janvier) pregnant mice were used and the presence of a vaginal plug was considered as embryonic day 0.5 (E0.5). The aspartame group received water with 0.015% aspartame (Sigma Aldrich, #A5139) until pups were postnatal day 1.5 (P1.5). On average, mice consumed 6.51 ± 0.29 ml (mean ± SD) of water with dissolved aspartame, which corresponds to 37 mg/kg. The EFSA-daily approved dosage for human intake per day is 40 mg/kg, the mouse equivalent of this dosage is 615 mg/kg using allometric scaling based on body surface^49^. Sex of pups (P1.5) was not determined, and the pups were randomly allocated to each experimental group.

### Tissue preparation

Mouse pups at P1.5 were subjected to hypothermia and brains were collected in 4% paraformaldehyde at room temperature (RT) and fixed overnight at +4°C. The brains were washed with PBS followed by transferring to 30% sucrose overnight at +4°C. The brains were embedded in OTC (Sakura Finetek) and then frozen in liquid N_2_. Coronal sections 40 μm thick were prepared using a cryostat (NX70, Epredia), mounted onto microscope slides (Superfrost Plus Adhesion Microscope slides, Epredia, J1820AMNZ), and stored at −30°C until further use.

### Immunohistochemistry

Immunohistochemistry was performed as described previously (Namba et al., 2020). Sections underwent antigen retrieval with 0.01 M citrate buffer (70°C, 60 min), followed by a cool down at RT (20 min), permeabilization in 1% Triton X-100/PBS, quenching in 0.1 M Glycin/PBS (30 min), and blocking with TX buffer (30 min, 0.2% gelatin, 300 mM NaCl, 0.3% Triton X-100 in PBS) at RT. Subsequently, slices were incubated with mouse anti-Satb2 (1:200, Abcam, ab5150), rat anti-Ctip2 (1:200, Abcam, ab18465), rabbit anti-TBR1 (1:200, Abcam, ab31940-100), rabbit anti-GAD65/67 (1:1000, Sigma, G5163), mouse anti-Olig2 (1:200, Chemicon, MABN50), rabbit anti-Olig2 (1:300, Cell signaling, #65915), rabbit anti-S100B (1:500, Swant, #37), rabbit anti-Ki67 (1:500, CST, #9129), goat anti-Sox10 (1:50, R&D, AF2864), and rabbit anti-cleaved caspase-3 (1:250, Cell Signaling #9661) primary antibodies in TX buffer at 4°C overnight. Afterwards, slices were washed in PBS (3 times), TX buffer for 30 min, and incubated with donkey anti-mouse Alexa 647 (1:500, Jackson, 715-605-151), anti-rat Cy3 (1:200, Jackson, 712-165-153), anti-chicken Alexa 488 (1:500, Jackson, 703-545-155), anti-rabbit Alexa 488 (1:500, Jackson, 711-545-152), anti-goat Cy3 (1:200, Jackson, 705-165-147), anti-rat Alexa 488 (1:500, Jackson, 712-165-153), and anti-rabbit Alexa 647 (1:500, Jackson, 711-605-152) secondary antibodies, followed by Hoechst 33342 (1:1000, Invitrogen) for 60 min at RT. Slices were washed with PBS and mounted with Mowiol mounting media.

### Image acquisition and quantifications

Fluorescent image acquisition was performed with the Zeiss LSM 980 single-photon scanning confocal microscope with the C-Apochromat 40x/1.20 W Korr water objective. Fluorescent images were taken as stacks with 0.33 μm optical sections. All images were analyzed and processed with ImageJ (http://imagej.nih.gov/ij/) using the Cell counter plugin (https://imagej.net/plugins/cell-counter). All quantifications were performed blindly from Z-projects with maximum intensity projection type and results were plotted in Prism (GraphPad Software).

### Statistical analysis

All statistical analysis was performed using GraphPad Prism 10 (GraphPad Software Inc, USA). All data is shown as mean ± SD. P-values less than 0.05 were considered statistically significant. The sample size was determined based on similar published papers in the field. Sample sizes (number of pups and dams) are indicated in the figure legends. Shapiro–Wilk normality test was used to assess the sample normality distribution. If the criteria for normal distribution were fulfilled, statistical significance was determined by unpaired Student’s *t*-test. If the criteria for the sample normality distribution were not met, statistical significance was determined by Mann–Whitney U test.

## Supplementary information

Supplementary Figure 1

## Data Availability

All data will be shared upon reasonable request.

## References

[1] Catalano, P. M. The impact of gestational diabetes and maternal obesity on the mother and her offspring. *J. Dev. Orig. Health Dis.* **1**, 208–215 (2010). 25141869 10.1017/S2040174410000115 PMC6691723

[2] Muglia, L. J., Benhalima, K., Tong, S. & Ozanne, S. Maternal factors during pregnancy influencing maternal, fetal, and childhood outcomes. *BMC Med.* **20**, 418 (2022). 36320027 10.1186/s12916-022-02632-6 PMC9623926

[3] Donato, J. Jr Programming of metabolism by adipokines during development. *Nat. Rev. Endocrinol.* **19**, 385–397 (2023). 37055548 10.1038/s41574-023-00828-1

[4] Czarnecka, K. et al. Aspartame-true or false? Narrative review of safety analysis of general use in products. *Nutrients* **13**10.3390/nu13061957 (2021). 10.3390/nu13061957 PMC8227014 34200310

[5] Dooley, J. et al. No effect of dietary aspartame or stevia on pancreatic acinar carcinoma development, growth, or induced mortality in a murine model. *Front Oncol.* **7**, 18 (2017). 28232906 10.3389/fonc.2017.00018 PMC5298959

[6] EFSA. Scientific opinion on the re-evaluation of aspartame (E 951) as a food additive. *EFSA J.* **11**, 3496 (2013).

[7] Yokogoshi, H., Roberts, C. H., Caballero, B. & Wurtman, R. J. Effects of aspartame and glucose administration on brain and plasma levels of large neutral amino acids and brain 5-hydroxyindoles. *Am. J. Clin. Nutr.* **40**, 1–7 (1984). 6204522 10.1093/ajcn/40.1.1

[8] Gebremichael, B., Lassi, Z. S., Begum, M. & Zhou, S. J. Effect of perinatal consumption of low-calorie sweetener on maternal health: A systematic review and meta-analysis. *Clin. Nutr. ESPEN* **63**, 164–176 (2024). 38954514 10.1016/j.clnesp.2024.06.029

[9] Huang, S. Y. et al. Aspartame consumption during pregnancy impairs placenta growth in mice through sweet taste receptor-reactive oxygen species-dependent pathway. *J. Nutr. Biochem.* **113**, 109228 (2023). 36435291 10.1016/j.jnutbio.2022.109228

[10] Rakic, P. Evolution of the neocortex: a perspective from developmental biology. *Nat. Rev. Neurosci.* **10**, 724–735 (2009). 19763105 10.1038/nrn2719 PMC2913577

[11] Sun, T. & Hevner, R. F. Growth and folding of the mammalian cerebral cortex: from molecules to malformations. *Nat. Rev. Neurosci.* **15**, 217–232 (2014). 24646670 10.1038/nrn3707 PMC4107216

[12] Namba, T. & Huttner, W. B. Neural progenitor cells and their role in the development and evolutionary expansion of the neocortex. *Wiley Interdiscip. Rev. Dev. Biol.* **6**10.1002/wdev.256 (2017). 10.1002/wdev.256 27865053

[13] Britton, S. M. & Miller, M. W. Neuronal loss in the developing cerebral cortex of normal and bax-deficient mice: effects of ethanol exposure. *Neuroscience* **369**, 278–291 (2018). 29138110 10.1016/j.neuroscience.2017.11.013

[14] Rubert, G., Minana, R., Pascual, M. & Guerri, C. Ethanol exposure during embryogenesis decreases the radial glial progenitorpool and affects the generation of neurons and astrocytes. *J. Neurosci. Res.* **84**, 483–496 (2006). 16770775 10.1002/jnr.20963

[15] Miller, M. W. Limited ethanol exposure selectively alters the proliferation of precursor cells in the cerebral cortex. *Alcohol Clin. Exp. Res.* **20**, 139–143 (1996). 8651443 10.1111/j.1530-0277.1996.tb01056.x

[16] Smiley, J. F. et al. Effects of neonatal ethanol on cerebral cortex development through adolescence. *Brain Struct. Funct.* **224**, 1871–1884 (2019). 31049690 10.1007/s00429-019-01881-1 PMC6565455

[17] Bird, C. W. et al. Binge-like ethanol exposure during the brain growth spurt disrupts the function of retrosplenial cortex-projecting anterior thalamic neurons in adolescent mice. *Neuropharmacology* **241**, 109738 (2023). 37778437 10.1016/j.neuropharm.2023.109738 PMC10842955

[18] Renwick, A. G. & Nordmann, H. First European conference on aspartame: putting safety and benefits into perspective. Synopsis of presentations and conclusions. *Food Chem. Toxicol.* **45**, 1308–1313 (2007). 17397982 10.1016/j.fct.2007.02.019

[19] Jones, S. K. et al. Transgenerational transmission of aspartame-induced anxiety and changes in glutamate-GABA signaling and gene expression in the amygdala. *Proc. Natl. Acad. Sci. USA* **119**, e2213120119 (2022). 36459641 10.1073/pnas.2213120119 PMC9894161

[20] Magnuson, B. A. et al. Aspartame: a safety evaluation based on current use levels, regulations, and toxicological and epidemiological studies. *Crit. Rev. Toxicol.* **37**, 629–727 (2007). 17828671 10.1080/10408440701516184

[21] Jones, S. K., McCarthy, D. M., Stanwood, G. D., Schatschneider, C. & Bhide, P. G. Learning and memory deficits produced by aspartame are heritable via the paternal lineage. *Sci. Rep.* **13**, 14326 (2023). 37652922 10.1038/s41598-023-41213-2 PMC10471780

[22] Alcamo, E. A. et al. Satb2 regulates callosal projection neuron identity in the developing cerebral cortex. *Neuron* **57**, 364–377 (2008). 18255030 10.1016/j.neuron.2007.12.012

[23] Kolk, S. M., Whitman, M. C., Yun, M. E., Shete, P. & Donoghue, M. J. A unique subpopulation of Tbr1-expressing deep layer neurons in the developing cerebral cortex. *Mol. Cell Neurosci.* **32**, 200–214 (2006). 16858776 10.1016/j.mcn.2005.08.022

[24] Arlotta, P. et al. Neuronal subtype-specific genes that control corticospinal motor neuron development in vivo. *Neuron* **45**, 207–221 (2005). 15664173 10.1016/j.neuron.2004.12.036

[25] Hevner, R. F. et al. Tbr1 regulates differentiation of the preplate and layer 6. *Neuron* **29**, 353–366 (2001). 11239428 10.1016/s0896-6273(01)00211-2

[26] Goldberg, E. M. & Coulter, D. A. Mechanisms of epileptogenesis: a convergence on neural circuit dysfunction. *Nat. Rev. Neurosci.* **14**, 337–349 (2013). 23595016 10.1038/nrn3482 PMC3982383

[27] Tremblay, R., Lee, S. & Rudy, B. GABAergic interneurons in the neocortex: from cellular properties to circuits. *Neuron* **91**, 260–292 (2016). 27477017 10.1016/j.neuron.2016.06.033 PMC4980915

[28] Lu, Q. R. et al. Sonic hedgehog-regulated oligodendrocyte lineage genes encoding bHLH proteins in the mammalian central nervous system. *Neuron* **25**, 317–329 (2000). 10719888 10.1016/s0896-6273(00)80897-1

[29] Zhou, Q., Wang, S. & Anderson, D. J. Identification of a novel family of oligodendrocyte lineage-specific basic helix-loop-helix transcription factors. *Neuron* **25**, 331–343 (2000). 10719889 10.1016/s0896-6273(00)80898-3

[30] Namba, T. et al. The fate of neural progenitor cells expressing astrocytic and radial glial markers in the postnatal rat dentate gyrus. *Eur. J. Neurosci.* **22**, 1928–1941 (2005). 16262632 10.1111/j.1460-9568.2005.04396.x

[31] Huang, H., He, W., Tang, T. & Qiu, M. Immunological markers for central nervous system glia. *Neurosci. Bull.* **39**, 379–392 (2023). 36028641 10.1007/s12264-022-00938-2 PMC10043115

[32] Porter, A. G. & Janicke, R. U. Emerging roles of caspase-3 in apoptosis. *Cell Death Differ.* **6**, 99–104 (1999). 10200555 10.1038/sj.cdd.4400476

[33] Haddad-Tovolli, R. & Claret, M. Metabolic and feeding adjustments during pregnancy. *Nat. Rev. Endocrinol.* **19**, 564–580 (2023). 37525006 10.1038/s41574-023-00871-y

[34] Sylvetsky, A. C. & Rother, K. I. Trends in the consumption of low-calorie sweeteners. *Physiol. Behav.* **164**, 446–450 (2016). 27039282 10.1016/j.physbeh.2016.03.030 PMC5578610

[35] Humphries, P., Pretorius, E. & Naude, H. Direct and indirect cellular effects of aspartame on the brain. *Eur. J. Clin. Nutr.* **62**, 451–462 (2008). 17684524 10.1038/sj.ejcn.1602866

[36] Trocho, C. et al. Formaldehyde derived from dietary aspartame binds to tissue components in vivo. *Life Sci.* **63**, 337–349 (1998). 9714421 10.1016/s0024-3205(98)00282-3

[37] Rycerz, K. & Jaworska-Adamu, J. E. Effects of aspartame metabolites on astrocytes and neurons. *Folia Neuropathol.* **51**, 10–17 (2013). 23553132 10.5114/fn.2013.34191

[38] Magnuson, B. A., Carakostas, M. C., Moore, N. H., Poulos, S. P. & Renwick, A. G. Biological fate of low-calorie sweeteners. *Nutr. Rev.* **74**, 670–689 (2016). 27753624 10.1093/nutrit/nuw032

[39] Xing, L. et al. Functional synergy of a human-specific and an ape-specific metabolic regulator in human neocortex development. *Nat. Commun.* **15**, 3468 (2024). 38658571 10.1038/s41467-024-47437-8 PMC11043075

[40] Peter G. Wells, G. P. M., Lutfiya Miller, Michelle Siu, J. Nicole Sweeting. *Oxidative Stress and Species Differences in the Metabolism, Developmental Toxicity, and Carcinogenic Potential of Methanol and Ethanol*. 169-253 (Wiley, 2013).

[41] Cai, J. et al. A crucial role for Olig2 in white matter astrocyte development. *Development* **134**, 1887–1899 (2007). 17428828 10.1242/dev.02847

[42] Liu, W. et al. Disruption of neurogenesis and cortical development in transgenic mice misexpressing Olig2, a gene in the Down syndrome critical region. *Neurobiol. Dis.* **77**, 106–116 (2015). 25747816 10.1016/j.nbd.2015.02.021 PMC4428323

[43] Bachmanov, A. A., Tordoff, M. G. & Beauchamp, G. K. Sweetener preference of C57BL/6ByJ and 129P3/J mice. *Chem. Senses* **26**, 905–913 (2001). 11555485 10.1093/chemse/26.7.905 PMC3718299

[44] Sclafani, A. & Abrams, M. Rats show only a weak preference for the artificial sweetener aspartame. *Physiol. Behav.* **37**, 253–256 (1986). 3737735 10.1016/0031-9384(86)90228-3

[45] Palatnik, A., Moosreiner, A. & Olivier-Van Stichelen, S. Consumption of non-nutritive sweeteners during pregnancy. *Am. J. Obstet. Gynecol.* **223**, 211–218 (2020). 32275895 10.1016/j.ajog.2020.03.034

[46] Di Bella, D. J. et al. Molecular logic of cellular diversification in the mouse cerebral cortex. *Nature* **595**, 554–559 (2021). 34163074 10.1038/s41586-021-03670-5 PMC9006333

[47] Yang, J. W. et al. Development of the whisker-to-barrel cortex system. *Curr. Opin. Neurobiol.* **53**, 29–34 (2018). 29738998 10.1016/j.conb.2018.04.023

[48] Ohte, N. et al. Differential neurogenic patterns underlie the formation of primary and secondary areas in the developing somatosensory cortex. *Cereb Cortex* **35**10.1093/cercor/bhae491 (2025). 10.1093/cercor/bhae491 PMC11795310 39756431

[49] Nair, A. B. & Jacob, S. A simple practice guide for dose conversion between animals and human. *J. Basic Clin. Pharm.* **7**, 27–31 (2016). 27057123 10.4103/0976-0105.177703 PMC4804402

