# Supplementary material for: No indication of histological changes in embryonic somatosensory cortex development upon maternal aspartame consumption in mice
Source: NPJ Sci Food. 2026 Jan 8;10:32. doi: 10.1038/s41538-025-00679-2 (PMC12867967; doi:10.1038/s41538-025-00679-2)
Supplement: Supplementary file 1 — Supplementary Figure 1 [file 41538_2025_679_MOESM1_ESM.docx]

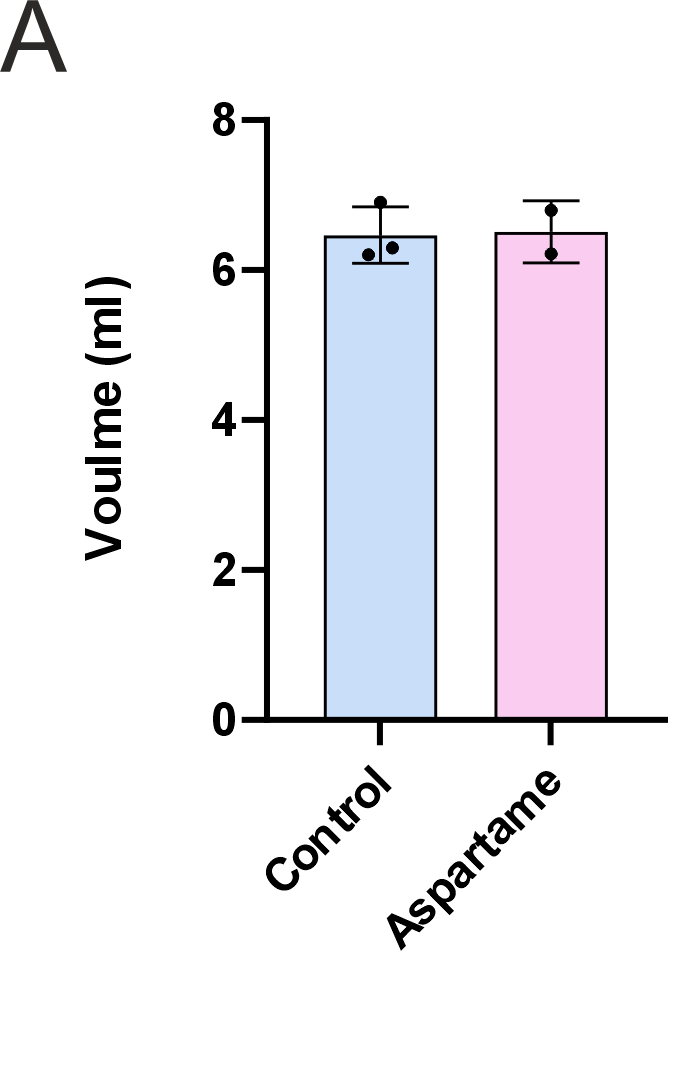


Supplementary Figure 1

(A) Water consumption of control (n=3 mice) and aspartame (n=2 mice) dams between E0.5-P1.5

Mean ± SD. Mann-Whitney U test.
